# Case Report: A rare case of primary graft failure after autologous stem cell transplant for mantle cell lymphoma rescued with darbepoetin, eltrombopag, and cyclosporine

**DOI:** 10.3389/frtra.2026.1811694

**Published:** 2026-04-15

**Authors:** Seav Huong Ly, Jordan Chung Cheng Hwang, Yeow Kheong Woon, Chandramouli Nargarajan, Jeffrey Kim Siang Quek, Yeh Ching Linn, Tertius T. Tuy

**Affiliations:** 1 Department of Haematology, Singapore General Hospital, Singapore, Singapore; 2 Division of Nursing, Singapore General Hospital, Singapore, Singapore

**Keywords:** autologous hematopoietic stem cell transplant, cyclosporine, darbepoetin, eltrombopag, mantle cell lymphoma, primary graft failure

## Abstract

We present a case of successful rescue of primary graft failure following autologous hematopoietic stem cell transplantation (autoHSCT) in mantle cell lymphoma using a combination of darbepoetin, eltrombopag, and cyclosporine. Primary graft failure is a rare but serious complication of HSCT that may lead to life-threatening infections, bleeding, and increased overall morbidity and mortality. The underlying mechanisms of graft failure in autoHSCT are not well elucidated, and management options are often limited to autologous stem cell boost or allogeneic stem cell transplantation. Our case highlights the use of a combination of darbepoetin, eltrombopag, and cyclosporine as a potential novel multipronged strategy for rescuing primary graft failure following autoHSCT.

## 1 Introduction

Mantle cell lymphoma is a subtype of mature non-Hodgkin B-cell lymphoma with a heterogeneous clinical course, ranging from indolent to aggressive disease (1). High-dose Ara-C-based chemoimmunotherapy regimens, followed by consolidative stem cell transplantation, are considered first-line treatment in young and medically fit patients (2).

Primary graft failure (PGF) is defined as the failure to achieve an absolute neutrophil count (ANC) ≥500/μuL by day +30, accompanied by pancytopenia (3, 4). The incidence of graft failure in HSCT is reported to be less than 3%–5% in autologous and matched allogeneic HSCT (alloHSCT) (5). PGF is often thought to be associated with alloHSCT; however, a few studies have reported that post-autoHSCT PGF occurs in approximately 1%–5% of cases (6, 7). Although an uncommon complication, PGF leads to severe sequelae, including fatal infections, bleeding, frequent blood transfusion requirements, and prolonged hospital stay (8–10). The pathophysiology of PGF following autoHSCT is not well understood, and no guidelines or consensus exist regarding its management. First-line treatment involves a stem cell boost or second transplantation (6, 7). However, when stem cells are not readily available, other medical modalities need to be explored. For example, eltrombopag is an oral thrombopoietin receptor agonist approved for severe aplastic anemia (11). Due to its proposed mechanism to stimulate trilineage hematopoiesis, eltrombopag has also been used to treat poor graft function following HSCT (12). Likewise, cyclosporine has been postulated to suppress autoreactive T lymphocytes and has been used anecdotally in a few cases of PGF (13–15).

In this case study, we describe a patient who developed PGF following autoHSCT and failed to respond to a stem cell boost. She was eventually salvaged with a combination of cyclosporine and growth factors—darbepoetin and eltrombopag.

## 2 Patient case

A 62-year-old woman initially presented with persistent and isolated thrombocytopenia, which was assumed to be immune thrombocytopenia (ITP), as she declined further evaluation. Her thrombocytopenia resolved spontaneously and she was lost to follow-up. Six years later, she re-presented with severe isolated thrombocytopenia and an increased immature platelet fraction of 17.3%. A preliminary bone marrow aspirate demonstrated increased megakaryopoiesis. Computed tomography (CT) of the neck, chest, abdomen, and pelvis showed two small (<1.7cm) isolated lymph nodes in the cervical and right inguinal lymph node regions. An excisional biopsy of the right inguinal lymph node was performed.

While awaiting results, her thrombocytopenia resolved after presumptive treatment with intravenous immunoglobulin (IVIg) and 0.5 mg/kg prednisolone, with platelet normalization by day 17 of treatment. Subsequently, her lymph node biopsy revealed mantle cell lymphoma with a Ki-67 index of approximately 20%. Bone marrow examination demonstrated a particulate aspirate that was moderately hypocellular for age, with trilineage hematopoiesis; flow cytometry showed no evidence of a clonal B-lymphocyte population, and trephine biopsy was negative for marrow involvement. Once mantle cell lymphoma was confirmed, she was initiated on the Nordic regimen and achieved a complete response at the end of treatment (16, 17).

Peripheral blood stem cell (PBSC) mobilization was performed after the fifth cycle of chemotherapy using granulocyte colony-stimulating factor (G-CSF). She mobilized 6.41 and $4.55\times 10^{6}$ CD34 cells/kg over 2 days, which were cryopreserved in Bag 1 and Bag 2, respectively. Following BEAM conditioning (BCNU, etoposide, cytosine arabinoside, and melphalan), she received an infusion of $6.41\times 10^{6}$ cells/kg of stem cells.

By day +21, she remained profoundly pancytopenic (Table 1), requiring frequent blood transfusions and experiencing several episodes of neutropenic sepsis, including *Streptococcus salvarius* bacteremia and pulmonary *Hormographiella aspergillata* infection. Hence, a decision was made to infuse the remaining $4.55\times 10^{6}$ cryopreserved CD34 cells/kg stem cells on day +21. Cell viability was assessed for both Bag 1 and Bag 2, which was found to be 80% for both bags.

**Table 1:** Treatments with white blood cells (10^9^/L), absolute neutrophil count (10^9^/L), hemoglobin (g/dL), and platelet count (10^9^/L) at different time points.

Day	0	+7	+14	+21	+30	+34	+40	+46
Treatment	PBSC			PBSC top-up		ESA + TPO-RA	CSA	
WBC	1.78	0.02	0.03	0.05	0.09	0.20	0.54	1.59
ANC	1.7	0	0	0	0	0	0	1.06
Hb	11	7.6	7.1	9.7	6.9	7.8	6.9	7.8
Plt	99	34	50	45	17	5	39	20

PBSC, peripheral blood stem cell; ESA, erythropoiesis-stimulating agent; TPO-RA, thrombopoietin receptor agonist; CSA, cyclosporine.

Despite the stem cell boost, her counts did not recover by day +30, meeting the criteria for PGF. CT imaging was unremarkable for relapsed mantle lymphoma, and repeat bone marrow aspirate and trephine biopsy showed marked trilineage hypocellularity, with no evidence of residual/relapsed mantle cell lymphoma. Evaluation for common infectious etiologies was negative, including cytomegalovirus (CMV), Epstein–Barr virus (EBV), human herpesvirus 6 (HHV-6), parvovirus, and there was no evidence of granuloma or fungal elements in the bone marrow.

**Figure 1:**
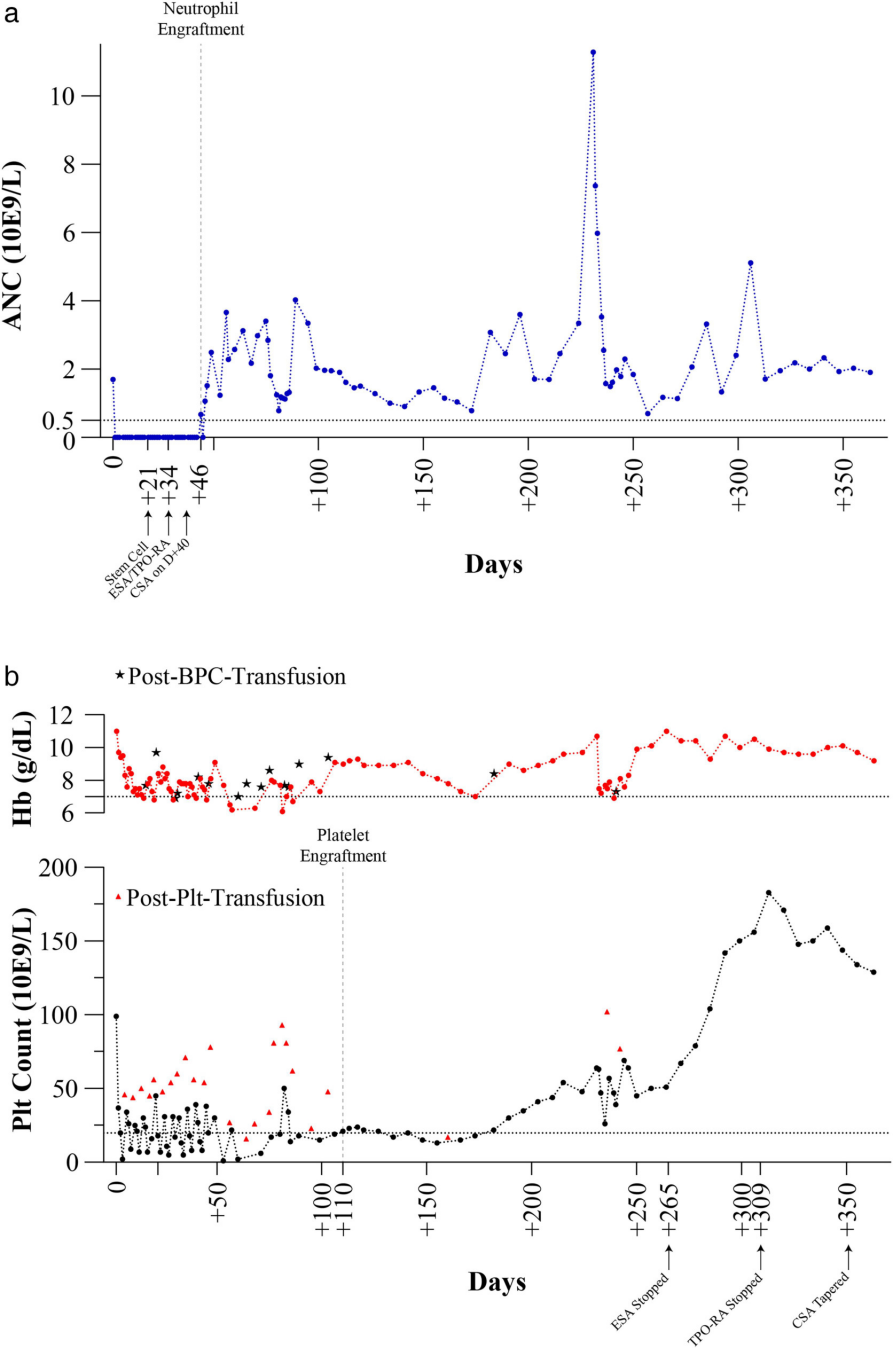
Blood indices over the span of 365 days demonstrating **(a)** absolute neutrophil recovery count and **(b)** hemoglobin and platelet recovery. Hemoglobin on the top in red and platelets on the bottom in black. ESA, erythropoiesis-stimulating agent; TPO-RA, thrombopoietin receptor agonist; CSA, cyclosporine.

On day +30, the patient was counseled and initiated on darbepoetin (120μg once per week) and eltrombopag (75 mg every morning) to synergistically boost hematopoietic recovery. However, by day +40, recovery remained inadequate. Despite the presence of an active invasive fungal infection, a shared decision was made with the patient to commence cyclosporine (1.5 mg/kg twice daily) to suppress autoreactive T lymphocytes and promote engraftment. After 6 days of cyclosporine, she achieved neutrophil engraftment on day +46 (Figure 1a). She was discharged on day +48 on continued therapy with darbepoetin, eltrombopag, and cyclosporine. Platelet engraftment was achieved on day +110 (Figure 1b). Darbepoetin was discontinued on day +265, eltrombopag was weaned off on day +309, and cyclosporine was tapered on day +351.

## 3 Discussion

Post-transplant hematopoietic outcomes can include normal engraftment, PGF, delayed engraftment, poor graft function, and secondary graft failure. The definitions of these outcomes are based on the consensus criteria established by four major groups, namely, the European Society for Blood and Marrow Transplantation (EBMT), the American Society for Transplantation and Cellular Therapy (ASTCT), the Center for International Blood and Marrow Transplant Research (CIBMTR), and the Asia-Pacific Blood and Marrow Transplantation (APBMT) (3, 4). These definitions are summarized in Table 2.

**Table 2:** Definitions of different post-transplant hematopoietic outcomes, including recovery and graft failure, based on consensus criteria from Sureda (2024) and Kharfan-Dabaja (2021).

Term	Definition
Neutrophil recovery	The first of 3 successive days with an ANC ≥500/μuL after post-transplantation nadir.
Platelet recovery	The first of 3 consecutive days with a platelet count of 20,000/μL or higher in the absence of platelet transfusion for 7 consecutive days.
Primary graft failure	Lack of achievement of an ANC ≥500/μuL by day +30 with associated pancytopenia (cell source: PBSC).
Secondary graft failure	A decline in hematopoietic function (may involve hemoglobin and/or platelets and/or neutrophils) necessitating blood products or growth factor support, after having met the standard definition of hematopoietic recovery.
Delayed engraftment	No consensus.
Poor graft function	Frequent dependence on blood and/or platelet transfusions and/or growth factor support in the absence of other explanations, such as disease relapse, drugs, or infections.

PBSC, peripheral blood stem cell.

Here, we present a rare case of PGF after autoHSCT in a patient with mantle cell lymphoma—who failed to achieve ANC ≥500/μuL by day +30. The pathophysiology of PGF following autoHSCT remains poorly understood. Factors that have been postulated to affect engraftment include stem cell dose and viability, intensity of the conditioning regimen, peri-transplant infections, and the presence of autoreactive T lymphocytes (18). For our patient, the post-thaw viability of cryopreserved stem cells was approximately 80%, and the total viable stem cell dose infused exceeded the minimum threshold of $2\times 10^{6}$ cells/kg. She received standard BEAM conditioning, and the most common viral infections (e.g., CMV, EBV, HHV6, and parvovirus) associated with engraftment impediment were ruled out. Although we were unable to discount the possibility of ongoing pulmonary *Hormographiella aspergillata* infection affecting her hematopoietic recovery, she was appropriately treated with antifungal therapy, and CT scanning showed stable disease. She eventually achieved neutrophil engraftment despite the presence of an active fungal infection.

In our patient, PGF developed despite an adequate stem cell boost on day +21. Capitalizing on the potential of eltrombopag to stimulate trilineage hematopoiesis, along with its promising success in treating poor graft function following HSCT, we combined darbepoetin and eltrombopag to achieve a synergistic enhancement of hematopoietic recovery.

The role of eltrombopag in primary graft failure has not been well described; however, its benefit in managing poor graft function after HSCT has been increasingly recognized. Tang et al. successfully treated 10 out of 12 patients with poor graft function following alloHSCT who were unresponsive to growth factors, mesenchymal stem cells, and decitabine, using eltrombopag initiated at 25 mg once daily and titrated up to 75 mg (19). Similarly, in a retrospective cross-sectional study, Kırcalı et al. demonstrated that 33 out of 39 patients with poor graft function after auto- or alloHSCT were successfully managed with eltrombopag and achieved transfusion independence (12). Eltrombopag is a small molecule that binds to the transmembrane domain of the thrombopoietin (TPO) receptor. Its effects extend beyond megakaryopoiesis, with demonstrated improvement of both erythropoiesis and myelopoiesis in severe aplastic anemia (11). Remarkably, the addition of eltrombopag to immunosuppressive therapy has been shown to improve overall response rates by nearly 50%, increase complete response rates by approximately twofold, and shortened the time to response to as early as 3 months (20). Eltrombopag promotes multilineage hematopoiesis via direct stimulation of hematopoietic stem and progenitor cells (HSPCs) through binding to the thrombopoietin receptor, MPL, which has downstream effects on myelopoiesis and erythropoiesis (21). In addition, Sun et al. demonstrated that eltrombopag leads to the expansion of bone marrow hematopoietic stem cells via JAK2/STAT5 signaling, thereby promoting multilineage hematopoiesis (22). Conversely, interferon-β has been reported to impair TPO-induced signaling pathways in HSPCs, resulting in HSPC depletion. Eltrombopag, by binding to a distinct site on MPL compared with TPO, bypasses interferon-β-mediated inhibition and thus improves the function of HSPCs (23). Finally, eltrombopag is a potent iron chelator; by reducing iron-induced reactive oxygen species, it may stimulate stem cell renewal via mechanisms independent of the thrombopoietin receptor (24).

By day +40, our patient’s hematopoietic recovery remained inadequate. Evidence regarding post-autoHSCT PGF, including its underlying mechanisms and optimal management, remains limited, with most data derived from case reports and small series. Kamble et al. reported the empirical use of cyclosporine (150 mg twice daily from day +28) to treat PGF following autoHSCT in a 44-year-old woman with anaplastic large-cell lymphoma; she achieved neutrophil recovery within 1 week (14). Cubillas et al. described five patients (three with multiple myeloma, one with composite lymphoma, and one with primary central nervous system lymphoma), of whom four developed PGF and one developed secondary graft failure following autoHSCT. These patients were unresponsive to increased G-CSF (all five), corticosteroids (four), or stem cell boost (two) but achieved neutrophil engraftment after empirical treatment with cyclosporine at 1.5 mg/kg twice daily (13). Likewise, in a case report by Shrinidhi et al., a 63-year-old woman with multiple myeloma developed persistent pancytopenia with a hypocellular bone marrow at day +24 following autoHSCT, for which she was empirically initiated on eltrombopag at 150 mg once daily in combination with cyclosporine at 1.5 mg/kg twice daily from day +26. She achieved an ANC ≥500 cells/uL by day +35 (15). Cyclosporine is an inhibitor of calcineurin, which is required for the activation of the transcription factor nuclear factor of activated T-cells (NFAT). By inhibiting calcineurin, cyclosporine prevents the synthesis of major cytokines, particularly interleukin-2 (IL-2), which is essential for T-cell activation and proliferation. Moreover, it has been postulated to suppress autoreactive T lymphocytes and was therefore used anecdotally in the above cases (13–15). Furthermore, cyclosporine may modulate bone marrow mesenchymal stem cells (MSCs), thereby improving the bone marrow microenvironment for hematopoiesis. Qu et al. investigated the effects of cyclosporine on the bone marrow niche in murine models and demonstrated that it promotes hematopoiesis while inhibiting MSC proliferation. Their findings suggest that cyclosporine suppresses adipogenic differentiation of MSCs, reduces inflammatory cytokines such as interleukin-6 (IL-6), and upregulates PD-L2 expression (25). Given the similarities between graft failure and severe aplastic anemia, as well as our patient’s prior history of ITP, we speculated that her autoimmune background raised the possibility of underlying immune dysregulation. Her graft failure may have been related to autoreactive T lymphocytes targeting hematopoietic stem cells. We hypothesized that cyclosporine—one of the frontline immunosuppressive therapies for severe aplastic anemia and a second-line agent for ITP—could play a beneficial role in salvaging her primary graft failure. Accordingly, on day +40, a shared decision was made with the patient to initiate cyclosporine at 1.5 mg/kg twice daily. By day +46, she achieved neutrophil recovery.

In summary, neutrophil engraftment in our patient was achieved after 6 days of cyclosporine treatment and 12 days of combined darbepoetin and eltrombopag therapy. Her white blood cell recovery and clinical course are detailed in Table 1 and Figure 1a. Within the limitations of this case report, we were unable to exclude the possibility of spontaneous recovery or to delineate the specific contribution of cyclosporine alone vs. its combination with eltrombopag and darbepoetin or the potential synergy between all three treatments. Nevertheless, we postulate that this combination may represent a multipronged strategy for rescuing PGF by suppressing autoreactive lymphocytes and enhancing trilineage hematopoiesis.

Further research is required to better understand the mechanisms and pathophysiology of PGF following autoHSCT, as well as to define the specific roles of cyclosporine, erythropoietin, and thrombopoietin receptor agonists.

## Data Availability

The original contributions presented in the study are included in the article/Supplementary Material, further inquiries can be directed to the corresponding author/s.
